# Supplementary material for: Trypanosome bloodstream-specific flagellum attachment proteins can mediate attachment in an insect surface coat environment
Source: J Cell Sci. 2026 Jan 13;139(1):jcs264370. doi: 10.1242/jcs.264370 (PMC12831198; doi:10.1242/jcs.264370)
Supplement: Supplementary information [file joces-139-264370-s1.pdf]

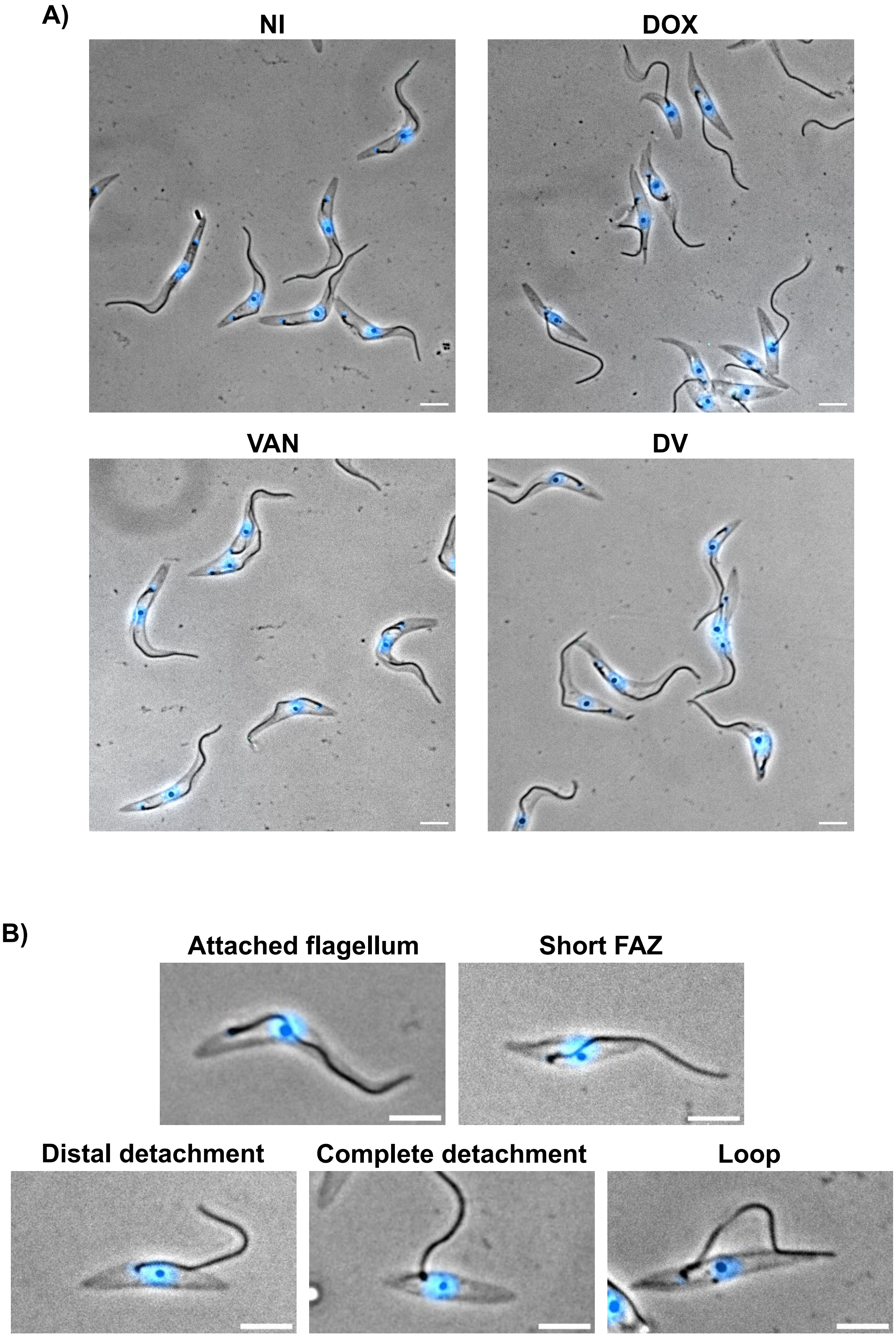

Fig. S1. A) Representative images of detergent-extracted cytoskeletons of FLA1-RNAi + FLA2 cell line after 24 hrs of induction. Non-induced cells (NI) or cells were induced with doxycycline (DOX), vanillic acid (VAN), or both doxycycline and vanillic acid (DV). **B)** Examples of flagellum attachment and detachment phenotypes. Cytoskeletons were extracted from cells following double-induction. Overlay of phase and Hoechst DNA (blue). Scale bar = 5 µm.

Journal of Cell Science • Supplementary information

A)

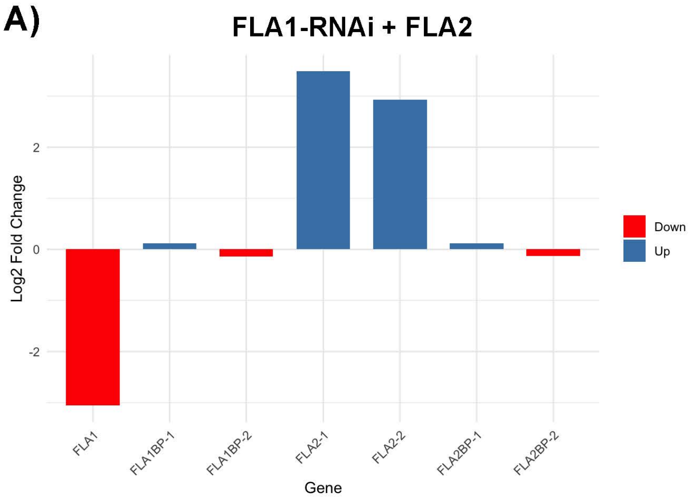

B)

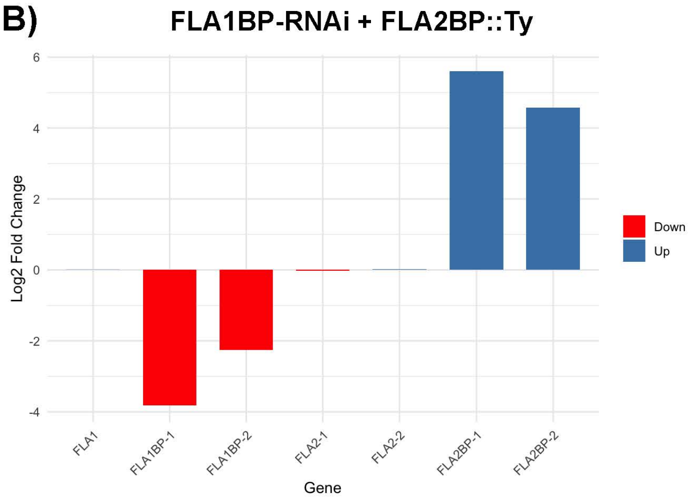

C)

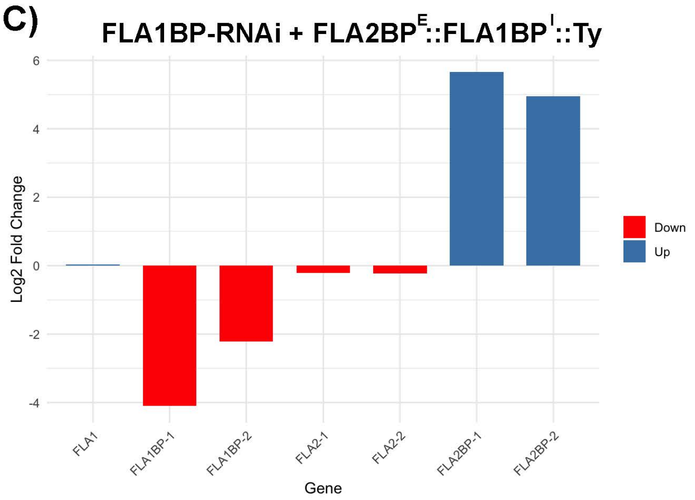

D)

Cell Line	Induction	Protein	Gene ID	RPKM
FLA1-RNAi + FLA2	NI	FLA2-1	Tb927.8.4060	3.90
FLA1-RNAi + FLA2	NI	FLA2-2	Tb927.8.4100	4.30
FLA1-RNAi + FLA2	DV	FLA2-1	Tb927.8.4060	79.70
FLA1-RNAi + FLA2	DV	FLA2-2	Tb927.8.4100	4.10
FLA1BP-RNAi + FLA2BP::Ty + FLA2	NI	FLA2-1	Tb927.8.4060	4.50
FLA1BP-RNAi + FLA2BP::Ty + FLA2	NI	FLA2-2	Tb927.8.4100	7.40
FLA1BP-RNAi + FLA2BP::Ty + FLA2	DV	FLA2-1	Tb927.8.4060	4.50
FLA1BP-RNAi + FLA2BP::Ty + FLA2	DV	FLA2-2	Tb927.8.4100	8.50
FLA1BP-RNAi + FLA2BPE::FLA1BPI::Ty + FLA2	NI	FLA2-1	Tb927.8.4060	10.30
FLA1BP-RNAi + FLA2BPE::FLA1BPI::Ty + FLA2	NI	FLA2-2	Tb927.8.4100	4.70
FLA1BP-RNAi + FLA2BPE::FLA1BPI::Ty + FLA2	DV	FLA2-1	Tb927.8.4060	9.20
FLA1BP-RNAi + FLA2BPE::FLA1BPI::Ty + FLA2	DV	FLA2-2	Tb927.8.4100	3.60

E)

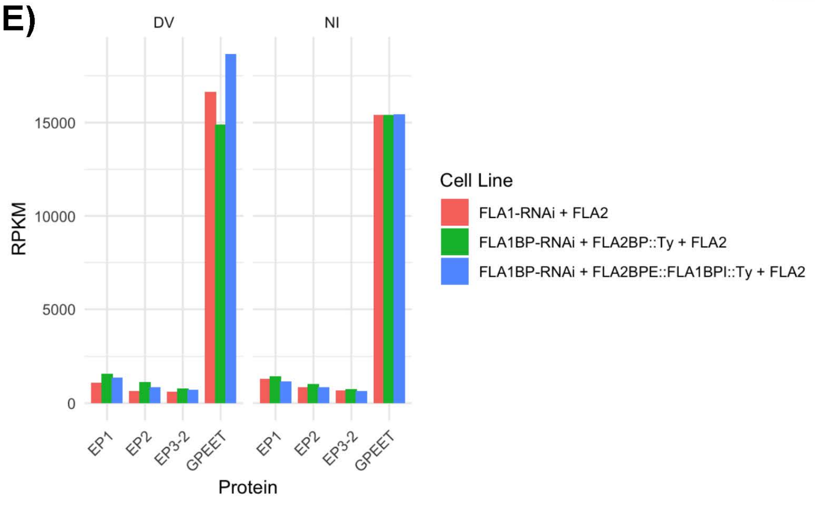

Fig. S2. Confirmation of FLA2 expression in the double-induction system. A-C) Changes in FLAs and FLABPs transcript levels based on mapping of all reads after the double-induction of the cell lines FLA1-RNAi + FLA2 (A), FLA1BP-RNAi + FLA2BP::Ty + FLA2 (B), and FLA1BP-RNAi + FLA2BPE::FLA1BPI::Ty + FLA2 (C). There are two near-identical copies of the genes which encode the proteins FLA2, FLA1BP and FLA2BP and these genes are designated FLA2-1, FLA2-2, FLA1BP-1, FLA1BP-2, FLA2BP-1, FLA2BP-2. In (A) FLA1 transcripts decrease while FLA2 transcripts decrease and in (B) and (C) FLA1BP transcripts decrease while FLA2BP transcripts increase. D) Uniquely mapped reads of FLA2-1 and FLA2-2 genes in non-induced (NI) and double-induced (DV) cell lines. Data from the NI FLA1-RNAi + FLA2 cell line shown was considered as the baseline. In the cell line FLA1BP-RNAi + FLA2BP::Ty + FLA2 the reads for FLA2-2 have doubled over the baseline, while in the cell line FLA1BP-RNAi + FLA2BPE::FLA1BPI::Ty + FLA2 the reads for FLA2-1 have doubled over the baseline. The gene IDs are from the TriTrypDB. RPKM - reads per kilobase of transcript per million reads mapped. E) Uniquely mapped reads of EP1 (Tb927.10.10260), EP2 (Tb927.10.10250), EP3-2 (Tb927.6.520) and GPEET (Tb927.6.510) procyclins in non-induced (NI) and double-induced (DV) cell lines.

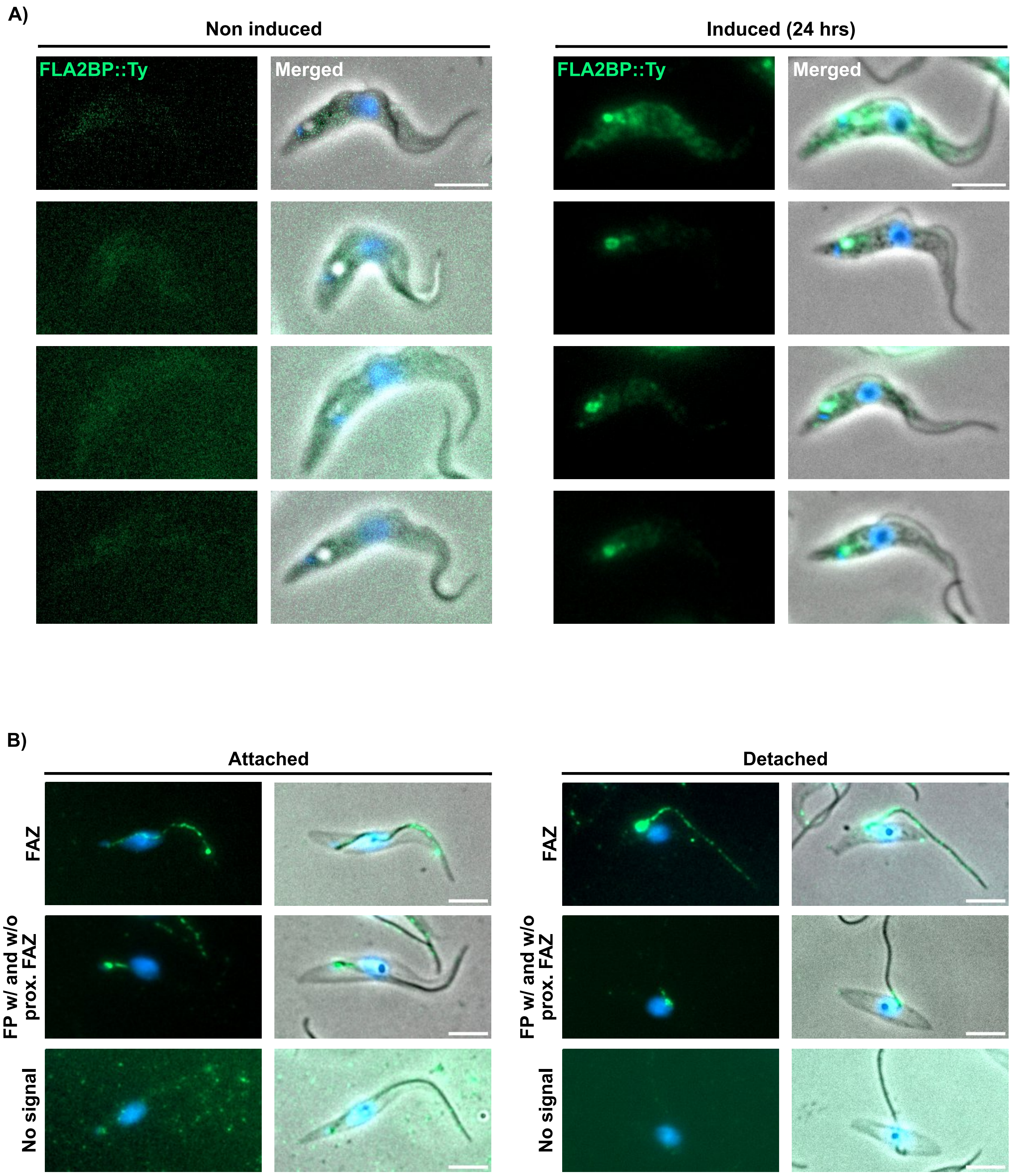

Fig. S3. A) Representative images of whole cells, induced with vanillic acid and fixed with paraformaldehyde. Overlay of phase, FLA2BP::Ty (green) and Hoechst DNA (blue). Scale bar = 5 µm. **B)** Representative images of detergent-extracted cytoskeletons with the different localisation categories of FLA2BP::Ty and FLA2BPE::FLA1BPI::Ty proteins observed in cells with an attached or detached flagellum. Overlay of phase, FLA2BP::Ty or FLA2BPE::FLA1BPI::Ty (green) and Hoechst DNA (blue). FP w/ and w/o prox. FAZ - flagellar pocket with and without proximal FAZ. FAZ protein localisation in parasites with detached flagellum reflects FLABP ability to access the entire flagellum, not being restricted to the flagellar pocket and proximal FAZ. Scale bar = 5 µm.

Journal of Cell Science • Supplementary information

Table S1.

Available for download at
https://journals.biologists.com/jcs/article-lookup/doi/10.1242/jcs.264370#supplementary-data

Journal of Cell Science • Supplementary information
